# Establishment of a tear protein biomarker panel differentiating between Graves’ disease with or without orbitopathy

**DOI:** 10.1371/journal.pone.0175274

**Published:** 2017-04-18

**Authors:** Cecilie Aass, Ingrid Norheim, Erik Fink Eriksen, Ellen Charlotte Børnick, Per Medbøe Thorsby, Milaim Pepaj

**Affiliations:** 1 Hormone Laboratory, Department of Medical Biochemistry, Oslo University Hospital, Oslo, Norway; 2 Faculty of Medicine, Institute of Clinical Medicine, University of Oslo, Oslo, Norway; 3 Deptartment of Endocrinology, Morbid Obesity and Preventive Medicine, Oslo University Hospital, Oslo, Norway; 4 University Department of Ophthalmology, Oslo University Hospital, Oslo, Norway; Universidade do Porto Faculdade de Medicina, PORTUGAL

## Abstract

**Background:**

Graves’ orbitopathy (GO) is an autoimmune inflammatory ocular complication and one of the most frequent manifestations of Graves’ disease (GD). Clinical judgment of GO is subjective sometimes leading to clinical and therapeutic challenges. Better tools to diagnose this severe complication are warranted.

**Patients and methods:**

The aim of the present study was to evaluate tear levels of LYZ, LACRT and AZGP1 in GD patients with or without GO, as possible biomarkers for GO. Tear samples were collected from GD patients with moderate-to-severe GO (n = 21) and no clinical signs of GO (n = 21). Additionally, 18 GD patients with mild GO and 9 patients without GO were included in a further part of the study.

**Results:**

Tear levels of LYZ (p < 0.001), LACRT (p = 0.004) and AZGP1 (p = 0.001) were significantly elevated in GD patients with moderate-to-severe GO compared to GD patients without GO. The discriminatory power of the three biomarkers, combined in a panel was confirmed by ROC plot analysis, with an AUC value of 0.93 (sensitivity of 95%; specificity of 80%). Since LYZ showed the best performance in discriminating between GD patients with (moderate-to-severe) and without GO (in combination with limited sample volume available), LYZ levels were also measured in tears from GD patients with mild GO and without GO. Significantly higher levels of LYZ were measured in GD patients with mild GO compared to those without GO (p = 0.003).

**Conclusions:**

We have established a novel three-protein biomarker panel that is able to discriminate between GD patients with and without GO, which might aid in diagnostic evaluation of GO as well as an indicator for disease activity.

## Introduction

Graves’ orbitopathy (GO) is an inflammatory autoimmune disorder of the orbit and one of the most frequent manifestations of Graves’ disease (GD) [1, 2]. GD is a common disorder that occurs more frequently in women than in men, with a peak age between 30 and 50 years [3–5]. GO is clinically apparent in approximately 20% of patients with GD [4, 6], and is characterized by inflammatory changes and enlargement of extraocular muscles and orbital tissues. These changes, combined with the local production of cytokines and other mediators of inflammation, result in upper eyelid retraction, edema, proptosis, diplopia, erythema of the perorbital tissues and conjunctivae, and even sight loss due to compressive optic neuropathy or breakdown of cornea [7, 8]. Generally, increased levels of thyrotropin receptor autoantibodies (TRAb) are considered as an important contributor to GO [9]. The symptoms of GO are primarily due to inflammation, and the severity of GO is categorized into mild, moderate, and severe (including sight-threatening) GO [10]. Although a general consensus for assessment of GO in routine clinical practice is established, according to the 2016 recommendations of the European Group on Graves’ Orbitopathy [10], the clinical judgment is subjective sometimes leading to clinical and therapeutic challenges. Therefore, from a clinical perspective, it is important to find disease-associated markers that identify GO patients.

In an earlier comprehensive quantitative proteomics study [11], we demonstrated increased levels of lysozyme C (LYZ), lacritin (LACRT) and zinc-alpha-2 glycoprotein 1 (AZGP1) in pooled tear fluid sample from GD patients with moderate-to-severe GO compared with GD patients without clinical signs of GO. The aim of the present study was therefore to assess individual levels of LYZ, LACRT and AZGP1 in tears from GD patients with moderate-to-severe GO and without GO using ELISA, and to investigate the diagnostic performance of these proteins on GO, either alone or combined in a panel.

## Materials and methods

### Subjects

As described in detail previously [11], tear samples from 21 patients with GD and moderate-to-severe GO (15 female, 6 male, median age 57 years, range 20–77 years; 9 smokers) were included in this study (moderate-to-severe GO, Table 1). Another group consisting of 21 patients with outburned GD and no sign of ocular symptoms during the disease (17 female, 4 male, median age 44 years, range 26–69 years; 3 smokers) served as the control group (Table 1) [11]. Patients in this group were examined by an endocrinologist (by the same endocrinologist as in the GO group), had normal TRAb values (except for two patients) and no history of GO [11].

**Table 1:** Demographic, clinical and serological data of GD patients with moderate-to-severe GO and without GO.

	Moderate-to-severe GO (n = 21)	Without GO (n = 21)	
Females, n (%)	15 (71.4)	17 (81)	
Males, n (%)	6 (28.6)	4 (19)	
Age, y (median)	57 (20–77)	44 (26–69)	
Smokers, n (%)	9 (42.8)	3 (14.3)	
Duration GD, month (median)	14 (3–108)	21 (10–35)	0.018
TRAb, lU/L (reference range, <1.8)	19.9 (3.7–156.4)	<0.9 (<0.9–4.9)	<0.001
Radioiodine treatment, n (%)	5 (23.8)	0 (0)	0.019
Thyroid surgery, n (%)	1 (4.8)	1 (4.8)	1
TSH, mlU/L (median; reference range, 0.5–3.6)	0.05 (0.01–1.7)	0.88 (0.03–4.34)	<0.001
Serum FT4, pmol/L (reference range, 8–21)	17.3 (10.1–49.2)	13.5 (11.4–21.3)	0.004
Serum FT3, pmol/L (reference range, 3.6–8.3)	7.4 (4.4–20.0)	5.4 (4.5–8.6)	0.005

Another study group consisting of 18 patients with GD and mild GO (15 female, 3 male, median age 51.5 years, range 28–63 years; 8 smokers) was also included in this study (mild GO, Table 2). Due to limited sample volume available, a new control group consisting of 9 patients with outburned GD and no sign of ocular symptoms during the disease (5 female, 4 male, median age 45 years, range 27–68 years; 2 smokers) served as the control group in this further part of the study (mild GO, Table 2). Patients in this group were examined by an endocrinologist (by the same endocrinologist as in the GO groups), had normal TRAb values (except for two patients) and no history of GO.

**Table 2:** Demographic, clinical and serological data of GD patients with mild GO and without GO.

	Mild GO (n = 18)	Without GO (n = 9)	
Females, n (%)	15 (83.3)	5 (55.6)	
Males, n (%)	3 (16.7)	4 (44.4)	
Age, y (median)	51.5 (28–63)	45 (27–68)	
Smokers, n (%)	8 (44.4)	2 (22.2)	
Duration GD, month (median)	8.5 (3–40)	18 (7–42)	0.047
TRAb, lU/L (reference range, <1.8)	5.4 (0.9–30.0)	<0.9 (<0.9–4.7)	0.003
Radioiodine treatment, n (%)	1 (5.5)	0 (0)	0.48
Thyroid surgery, n (%)	2 (11.1)	0 (0)	0.31
TSH, mlU/L (median; reference range, 0.5–3.6)	0.04 (0.03–1.7)	0.1 (0.03–2.33)	0.65
Serum FT4, pmol/L (reference range, 8–21)	21.3 (12.2–28.2)	18.6 (11.4–26.1)	0.37
Serum FT3, pmol/L (reference range, 3.6–8.3)	6.3 (4.6–11.7)	6.2 (4.8–8.6)	0.93

All GO subjects went through a thorough examination by a trained ophthalmologist and an endocrinologist, and filled out a questionnaire regarding clinical history (other autoimmune,—and ocular diseases), smoking habits, symptoms and medications. However, ocular surface diseases, such as dry, which may alter the tear composition, cannot be completely excluded since GD and GO patients may have dry eyes. None of the patients with GO had started on medical treatment with steroids (which may affect the ocular disease). A clinical score (mild, moderate and severe) was given by the ophthalmologist to all the patients with GO, based on their overall severity (eyelid swelling, eyelid aperture, proptosis, eye motility, visual acuity and color vision) [12] and clinical activity, at the time of sample collection. All GO patients were in the active phase of the disease course, with a CAS score ≥ 3/10 (10 when severity is included). Written informed consent was obtained from all the subjects and all protocols were approved by Regional Committee for Medical and Health Research Ethics (REK south-east, 2013/839) in accordance with the ethical standards laid down in the Declaration of Helsinki [11, 13].

### Materials, tear collection and protein extraction

Materials, tear collection and protein extraction are described in detail previously [11] with some alterations. See details in S1 File.

### ELISA measurements

One Schirmer strip from each individual was extracted as described in S1 File and the concentration of the three proteins was assessed with commercially available ELISA kits; AZGP1 (detection range; 4.7–300 ng/ml), LACRT (detection range; 1.56–100 ng/ml) and LYZ (detection range; 0.156–25 ng/ml). Additionally LYZ levels were also assessed in both mild GO group and controls. Protein concentrations obtained with ELISA assays were normalized with respect to total protein concentration and expressed as protein/total protein concentration in μg/mg.

### Statistical analysis

ELISA results were exported to SPSS statistical analysis program (IBM SPSS statistics 22, Armonk, NY, USA). Data are presented as median and interquartile range (IQR). We analyzed non-normally distributed data, log-transformed, or used non-parametric methods, as appropriate. For comparison of continuous variables between groups, student’s *t* tests or two-sided Mann-Whitney U tests were used. Spearman correlation coefficients rank test were used for TRAb correlations. A two-sided p-value < 0.05 was considered to be significant and uncorrected values are presented. Bonferroni-Holm correction was also performed. The diagnostic value for LYZ, LACRT and AZGP1, individually and together, was assessed by ROC-curve analyses. Multiple logistic regression analysis were performed with log-transformation of parameters when needed, to assess if there was any potential effects from independent variables such as age, smoking, gender and protein concentration on GO (dependent variable).

## Results

### Moderate-to-severe GO

#### Demographic, serological and clinical data

Twenty one GD patients with moderate-to-severe GO and 21 GD patients without signs of GO were included in the study, as shown in Table 1. The number of smokers was significantly higher in the moderate-to-severe GO group (p = 0.043) than those without GO, and the moderate-to-severe GO group were significantly older than those with no signs of GO (p = 0.024). The mean values for duration of GD, TRAb, TSH, serum T3 and serum T4 were significantly different between the groups (see Table 1 for p-values). Among the patients with moderate-to-severe GO, five had been treated with radioiodine and one had gone through thyroid surgery. On the other hand, none of the GD patients without GO were treated with radioiodine, while one had gone through thyroid surgery (Table 1).

#### Tear levels of LYZ, LACRT and AZGP1

**Fig 1:**
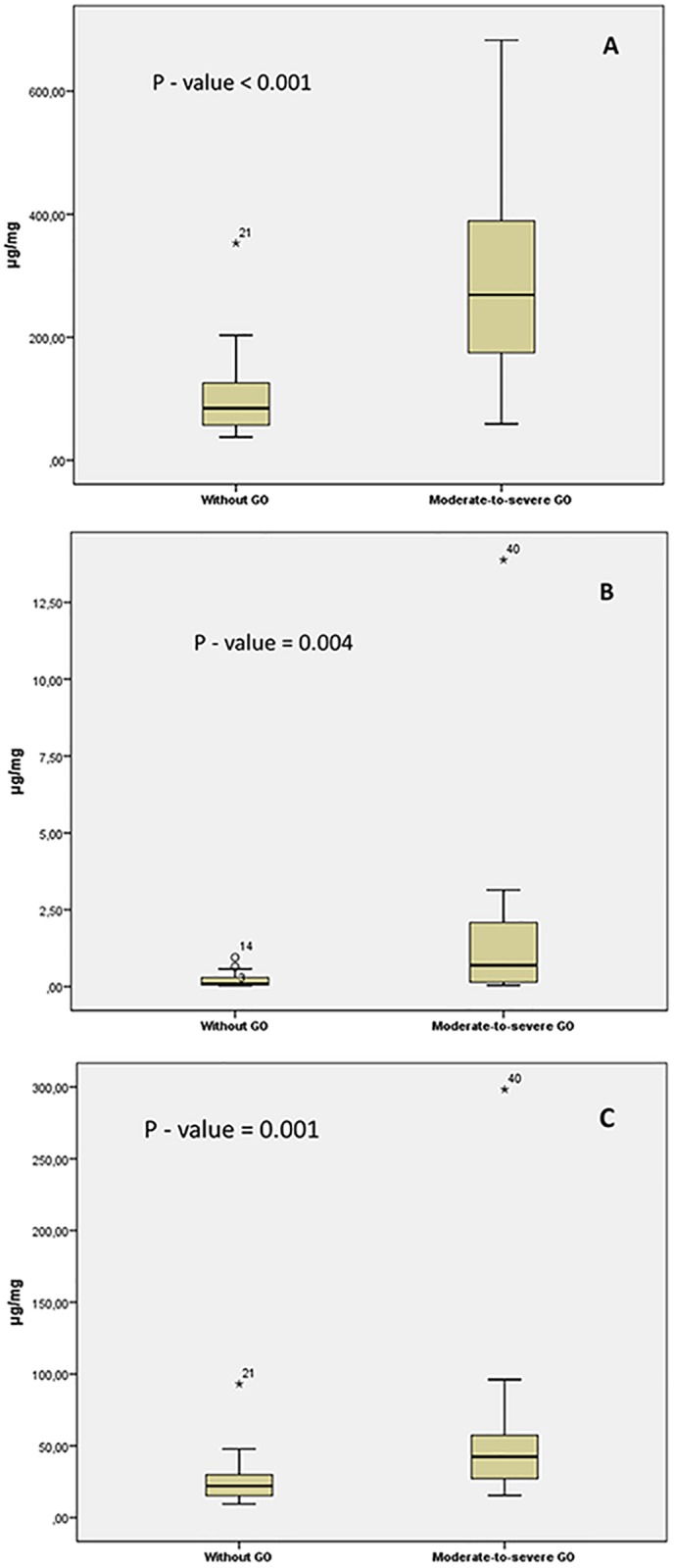
Tear levels of LYZ, LACRT and AZGP1 in moderate-to-severe GO patients vs GD patients without GO obtained with ELISA. Box and-whisker plot showing median and interquartile range (IQR). (A) The median for LYZ (268.9 μg/mg vs 84.3 μg/mg), (B) LACRT (6.9 μg/mg vs 0.9 μg/mg) and (C) AZGP1 (42.5 μg/mg vs 22.1 μ/mg). Mann-Whitney U-tests were performed and *p*-values are indicated on each graph.

We determined tear levels of LYZ, LACRT and AZGP1 with commercially available ELISA assays. The levels of LYZ ((median, 268.9 μg/mg (n = 21) vs. 84.3 μg/mg (n = 21), p < 0.001)), LACRT ((median, 6.9 μg/mg (n = 20) vs. 0.9 μg/mg (n = 20), p = 0.004)) and AZGP1 ((median, 42.5 μg/mg (n = 20) vs. 22.1 μ/mg (n = 20), p = 0.001)) were significantly higher in GD patients with moderate-to-severe GO compared to GD patients without GO, as shown in Fig 1A–1C. These results validate our previous findings, that levels of these three proteins are elevated in GD patients with moderate-to-severe GO [11]. Importantly, LACRT levels were detected in only 13 of 20 tear samples from the GO group and 11 of 20 tear samples from the control group, probably due to limited sample volume available. However, tear levels for the samples not detected with ELISA were set to the lowest obtained concentration (0.2 μg/mg) for ROC analysis.

Logistic regression analysis showed that elevated levels of LYZ (p = 0.001), LACRT (p = 0.006) and AZGP1 (p = 0.006) were significantly associated with GO (Table 3). Moreover, higher levels of LYZ, AZGP1 and LACRT were significantly associated with GO after adjustment for age, smoking and gender ((p = 0.002, OR = 10.53, 95% CI = (2.38, 46.58)), ((p = 0.016, OR = 4.99, 95% CI = (1.35, 18.57)) and ((p = 0.035, OR = 1.59, 95% CI = (1.03, 2.44)), respectively in a multivariable logistic regression analysis (Table 3). Increased tear levels of LYZ, AZGP1 and LACRT correlated with increased TRAb values in patients with moderate-to-severe GO (LYZ, p < 0.001, LACRT, p = 0.002, AZGP1, p < 0.001).

**Table 3:** Logistic regression analysis for moderate-to-severe GO patients in relation to LYZ, LACRT and AZGP1 both with and without adjustment for smoking, gender and age using log-transformed data.

	No adjusted effect (OR)	95% CI	p-value	Adjusted effect (OR)	95% CI	p-value
LYZ	8.90	2.58–30.70	0.001	10.53	2.38, 46.58	0.002
AZGP1	4.81	1.58–14.69	0.006	4.99	1.35, 18.57	0.016
LACRT	1.70	1.16–2.50	0.006	1.59	1.03, 2.44	0.035

**Fig 2:**
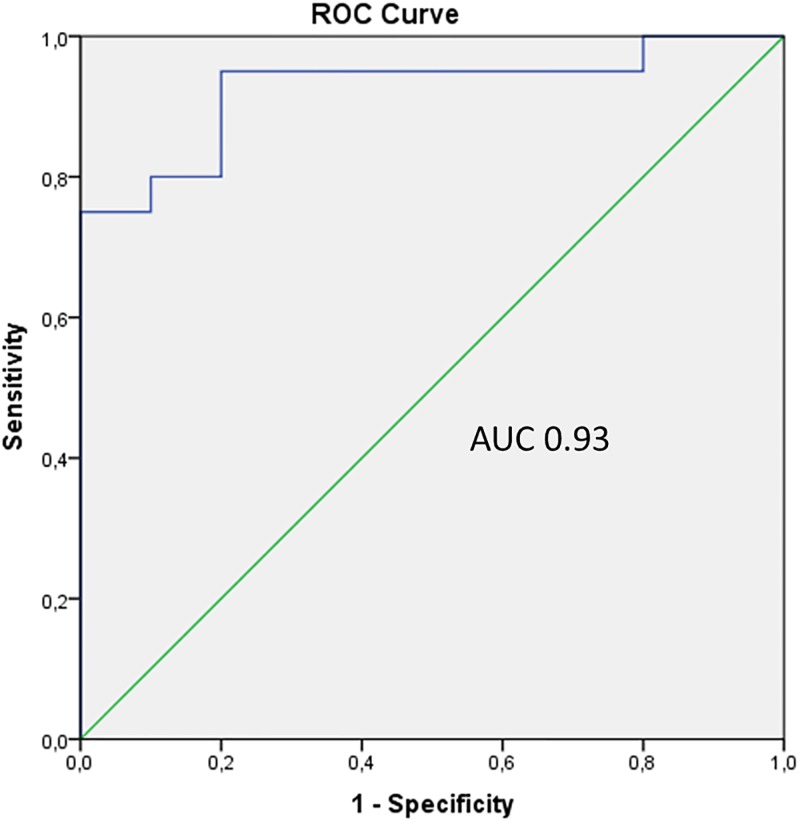
Diagnostic performance of the 3-protein biomarker panel in discriminating GD patients with moderate-to-severe GO from GD patients without GO. ROC curve analysis showing LYZ, LACRT and AZGP1 combined in a panel, with an AUC of 0.93. The curve is created by plotting the true positive rate (sensitivity) against the false positive rate (1 –specificity).

The diagnostic performance of LYZ, LACRT and AZGP1 was established by calculating area under the curves (AUC) representing receiver operator characteristic (ROC) plots. We first assessed their individual performance in discriminating between GD patients with and without GO. LYZ showed high accuracy with an AUC value of 0.91 (sensitivity of 95% and specificity of 71%), while LACRT and AZGP1 showed AUC values of 0,77 (sensitivity of 95% and specificity of 70%) and 0,80 (sensitivity of 95% and specificity of 75%), respectively. A better performance than any of the individual biomarkers was achieved when the biomarkers where combined into a panel, with an AUC value of 0.93 (sensitivity of 95%, specificity of 80%, p-value < 0.001, 95% CI = 0.83, 1.0), as shown in Fig 2.

### Mild GO

#### Demographic, serological and clinical data

In this further study, eighteen GD patients with mild GO and 9 GD patients without signs of GO (Table 2) were included. Notably, none of these individuals overlapped with the other study group. Due to limited sample volume available, a new group served as a control group in this further part of the study. Patients in the mild GO group were not significantly older than those with no signs of GO (p = 0.50), and the number of former and current smokers was not significantly higher in the mild GO group (p = 0.27). The mean values for duration of GD and TRAb were significantly different between the groups (see Table 2 for p-values). Moreover, among the patients with mild GO, one had been treated with radioiodine and two had gone through thyroid surgery. None of the GD patients without GO were treated with radioiodine or had gone through thyroid surgery (Table 2), and none of the patients with mild GO had started treatment with steroids at the time of sample collection.

#### Tear levels of LYZ

**Fig 3:**
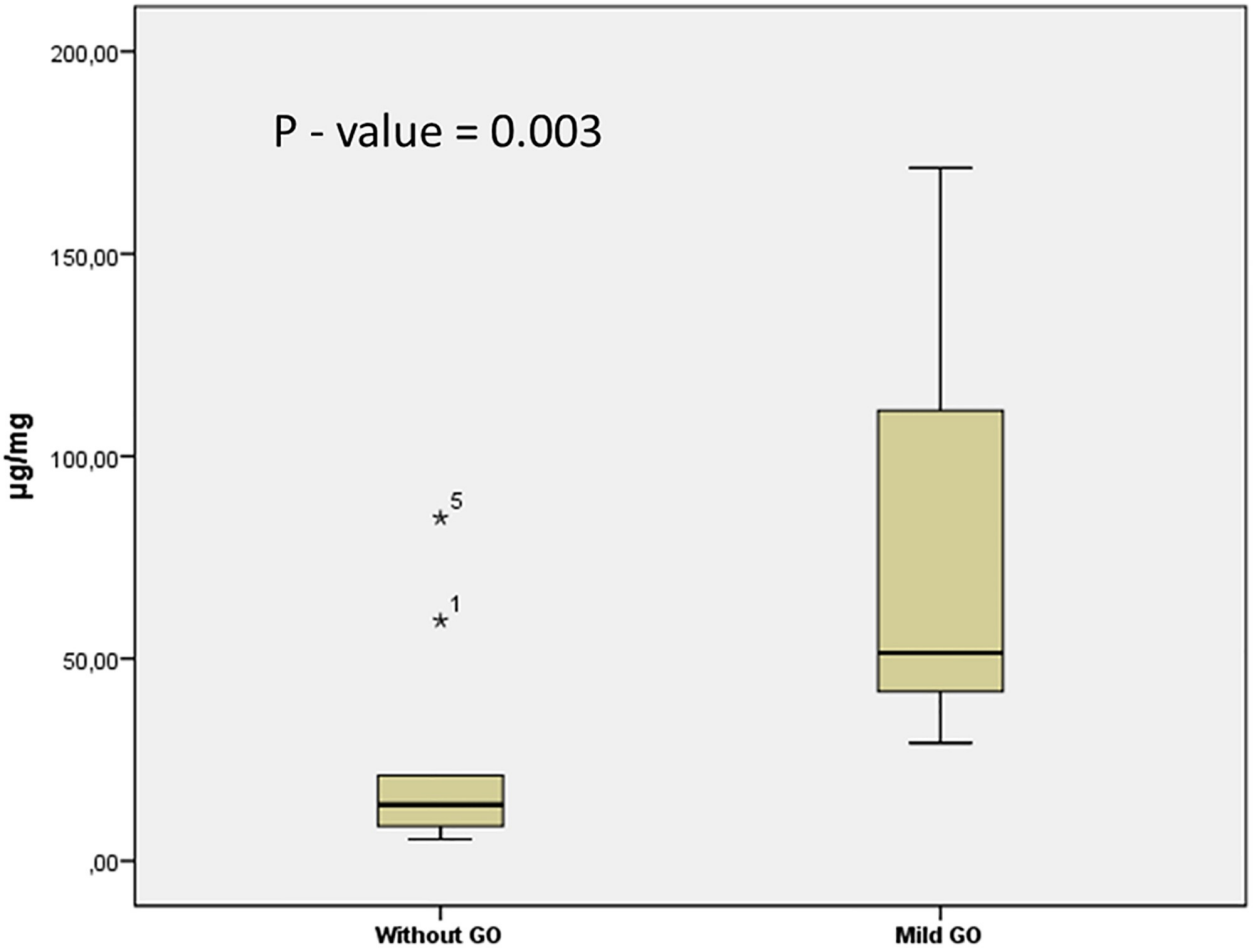
Tear levels of LYZ in mild GO patients vs GD patients without GO obtained with ELISA. Box and-whisker plot showing median and interquartile range (IQR). The median for LYZ (51.4 μg/mg vs. 13.9 μg/mg). Mann-Whitney U-test was performed and the *p*-value is indicated on the graph.

Since LYZ showed the best performance in discriminating between GD patients with moderate-to-severe GO and those with no signs of GO, we next tested whether GD patients with mild GO also showed a pronounced increase in LYZ levels compared to GD patients without clinical signs of GO. ELISA analysis showed that levels of LYZ (median, 51.4 μg/mg vs. 13.9 μg/mg, p = 0.003) were significantly higher in the mild GO group (Fig 3). Additionally, a ROC curve was generated, showing an AUC of 0.86 (sensitivity of 100% and specificity of 78%, S1 Fig). Notably, increased tear levels of LYZ did not correlate with TRAb levels (p = 0.57) in the mild GO group.

Using logistic regression analysis, elevated levels of LYZ were significantly associated (p = 0.010) with GO, and this association was still significant after adjustment for age, smoking and gender ((p = 0.032, OR = 7.58, 95%—CI = (1.19, 48.40)).

## Discussion

In our previous proteomic study [11], we showed that tear levels of LYZ, AZGP1 and LACRT were elevated in pooled samples of GD patients with moderate-to-severe GO compared to those without GO. The main reason why these three proteins were chosen was their high abundance in tear fluid, which is highly important when dealing with limited sample volume available, in combination with ELISA. The objective of this study was therefore to examine the protein levels of LYZ, LACRT and AZGP1 as potential protein biomarkers for GO and to validate our previous findings [11] using individual protein profiling.

### Moderate-to-severe GO

Out of the 21 patients with moderate-to-severe GO, smoking were more prevalent in the GO group compared to the control group (9 vs. 3), showing that about 40% of the GO patients smoke, which has also been suggested in previous studies [14]. Smoking is a well-established risk factor for developing GO, and the odds increase progressively as the severity of eye disease increases. It is believed that GD is caused by TRAb, which activate TSHR, and enhanced expression of the autoantigen TSHR within the orbit may play a role in the initiation or propagation of the autoimmune response in GO [15]. In this study, increased TRAb levels were observed in all patients with moderate-to-severe GO, and median TRAb levels were significantly higher in these patients in comparison to patients with no signs of GO. Although measurement of serum TRAb levels in patients with GO is recommended, assessment of activity and severity of GO are measured by the CAS and the features of eyelid swelling, eyelid aperture, proptosis, eye motility, visual acuity and color vision [12]. Thus, the clinical judgment in estimating the activity and severity of GO is highly subjective leading to major clinical and therapeutic challenges.

Tear levels of LYZ, a major lacrimal gland protein, were elevated in the GO group (Fig 1A). The present results with increased tear levels of LYZ in patients with GO corroborates with results observed in previous studies [11, 16, 17]. In logistic regression analysis, a significant association between GO and LYZ was obtained, both with and without adjustment for age, gender and smoking. Additionally, a significant correlation was observed between LYZ levels and TRAb values.

Moreover, tear levels of LACRT, a major lacrimal gland protein, were also elevated in the GO group (Fig 1B). Notably, LACRT levels were detected in only 13 of 20 tear samples from the GO group and 11 of 20 tear samples from the control group with ELISA assay, probably due to limited sample volume available. Further analyses of LACRT are necessary to support these current findings. Though well-characterized ELISA assays are suitable tools for verification analysis, sometimes sample volume needed is an issue, especially with tear fluid as only a few microliters are obtained from an individual. A highly viable alternative to ELISA is the use of targeted MS methods using multiple reaction monitoring (MRM) approaches. This methodology is highly suited for candidate verification as it is possible to quantify many proteins with high sensitivity in a single LC-MRM-MS run [18].

Similarly, AZGP1, a multidisciplinary tear protein [19], was found increased in the GO group (Fig 1C). These results are in line with previously reported up-regulation of AZGP1 in tears from patients with GO and healthy smokers [20]. In logistic regression analysis, a significant association between GO and AZGP1 was obtained, both with and without adjustment for age, gender and smoking. Since our groups are not matched for smoking, we cannot not rule out the possibility that smoking may affect the AZGP1 tear levels since smoking is a significant risk factor for the development of GO [11]. Although we did not find any correlation between AZGP1 levels and smoking in the GO group, additional studies with larger groups, matched for smoking, are necessary to determine the impact of smoking on AZGP1 tear level.

As mentioned earlier, clinically, it is very valuable for the ophthalmologist to have disease-associated markers to aid in diagnosis of GO. The results from this study have shown that it is possible to differentiate between GD patients with and without GO by measuring tear levels of LYZ, LACRT and AZGP1. As it is well-known that ocular diseases may alter the tear composition, it should be mentioned that the secretion of LYZ, LACRT and AZGP1 have been detected in tears of healthy subjects [21, 22]. Additionally, LYZ has also shown to be down-regulated in patients with dry-eye syndrome [23]. Although the diagnostic performance (ROC curve analysis) of the three proteins separately was satisfactory (reflected by high values for AUC), in a complex disease such as GO, it is difficult to discriminate patients using individual biomarkers. Hence, a combination of biomarkers rather than a single one likely performs better as a diagnostic test. When combined, LYZ, LACRT and AZGP1 form a powerful tear fluid panel able to discriminate between GD patients with moderate-to-severe GO and those without signs of GO with 93% accuracy. The diagnostic performance of our biomarker panel now needs to be further validated in a prospective longitudinal study with a larger population. Tear collection from such a cohort are underway in our lab.

### Mild GO

Since LYZ showed the best performance in discriminating between GD patients with moderate-to-severe GO and those with no signs of GO, and in combination with limited sample volume available, we next examined the ability of LYZ to distinguish between GD patients with mild GO from GD patients without clinical signs of GO. The levels of LYZ were significantly higher in GD patients with mild GO compared to GD patients without GO (Fig 3), representing a possible manifestation of subclinical stage of the disease. Moreover, LYZ was significantly associated with GO, and this association was still significant after adjustment for age, smoking and gender. With an AUC of 0.86, LYZ also show potential for differentiating GD patients with mild GO from those with no signs of GO. The difference between mean age and the number of smokers was not significantly different between the patients included in the mild GO group and patients with no signs of GO. However, increased TRAb levels were observed in 16 patients in the mild GO group, and median TRAb levels were significantly higher in this group compared to patients with no signs of GO.

Today, patients with mild GO are usually not recommended for anti-inflammatory treatment with steroids, however, many patients suffer from changes in their appearance and can have troublesome diplopia [24]. Generally, a wait-and-see policy is favored, in hope of a spontaneous improvement [24]. However, some of these patients progress to more moderate to severe stage of GO disease, representing a subgroup which cannot be detected with clinical examination methods. We believe that tear fluid measurement of LYZ may help the clinician in deciding which of the patients that needs extra care concerning development of GO in the clinically mild GO group.

## Concluding remarks

The aim of the present study was to assess levels of LYZ, LACRT and AZGP1 in tear fluid from GD patients with moderate-to-severe and without GO using ELISA, and to investigate the diagnostic performance of these proteins either alone or combined in a panel. We have established a novel three protein biomarker panel that is able to differentiate between GD patients with GO from those without GO. This diagnostic marker panel could enable non-invasive diagnosis and be of tremendous benefit for patients who are being evaluated for this debilitating disease. Additionally, LYZ measured in tears from GD patients with mild GO were significantly higher compared to GD patients without GO.

Since dry eye is common among patients with GD and GO, and the groups are not matched for age, gender and smoking we cannot rule out at this point, that the altered tear composition are only a result of GO. Furthermore, further evaluation of these biomarkers on a larger cohort of GD patients, and patients with other forms of hyperthyroidism, would be necessary to increase the diagnostic accuracy of the biomarker panel.

## Supporting information

S1 File Materials, tear collection, and protein extraction. (DOCX)

S1 Fig Diagnostic performance of LYZ in discriminating GD patients with mild GO from GD patients without GO. (TIFF)
