# Supplementary material for: Establishment of a tear protein biomarker panel differentiating between Graves’ disease with or without orbitopathy
Source: PLoS One. 2017 Apr 18;12(4):e0175274. doi: 10.1371/journal.pone.0175274 (PMC5395154; doi:10.1371/journal.pone.0175274)
Supplement: S1 File — (DOCX) [file pone.0175274.s001.docx]

**S1.1 Materials**

As described in detail previously [11], all chemicals and solvents were purchased from Sigma (St. Louis, MO, USA) unless stated otherwise. Milli Q water was obtained from a Milli-Q Ultrapure water purification system (Millipore, Bedford, MA, USA). Sodium chloride (NaCl) was purchased from Merck (Darmstadt, Germany). Bio-Rad protein assay dye reagent concentrate and ɣ- microglobulin standard were obtained from Bio-Rad Laboratories (Hercules, CA, USA). Cut-off size (0.1 µM) ultrafree-MC (0.5 mL) VV centrifugal filter used for the extraction of proteins was from Millipore (Bedford, MA, USA). Lysozyme C (detection range; 0,156-20 ng/ml) was obtained from abcam, Cambridge, UK. Zinc-alpha-2-glycoprotein 1 (detection range; 4,7-300 ng/ml) and lacritin (detection range; 1,56-100 ng/ml) were purchased from Cloud-clone corp., Houston, TX, USA.

**S1.2 Tear collection.**

As described in detail previously [11], tear samples were collected using Schirmer’s type I tear test (Haag-Streit UK Ltd, Harlow, Essex, UK) without using local anesthesia. The Schirmer strips were inserted for five minutes or until the strips were full (standardized collection time) in the lower eyelid in the standard fashion in both eyes (open eye tears). The same object carried out the collection of tears from all the subjects. After collection, the Schirmer strips were placed in a 2 ml microcentrifuge tube and immediately frozen at – 80 ^o^C until analysis.

**S1.3 Protein Extraction**

As described previously [11], one Schirmer strips from each patient was collected and placed in a 0.1 µm centrifugal filter unit. Tear proteins were then extracted from the Schirmer strips with 500 µL 100 mM triethylammonium bicarbonate (TEAB) and 50 mM NaCl, and mixed for 4 h at 25 ^o^C at 300 rpm. Next, the samples were centrifuged at 7500 rpm for 5 min and the Schirmer strips were removed. Total protein concentration measurements were performed by a colorimetric protein assay using a Microplate absorbance reader (Tecan Austria GmbH, Grӧdig, Austria) with ɣ-microglobulin as standard.
