# Supplementary figures and images for: Establishment of a tear protein biomarker panel differentiating between Graves’ disease with or without orbitopathy
Source: PLoS One. 2017 Apr 18;12(4):e0175274. doi: 10.1371/journal.pone.0175274 (PMC5395154; doi:10.1371/journal.pone.0175274)

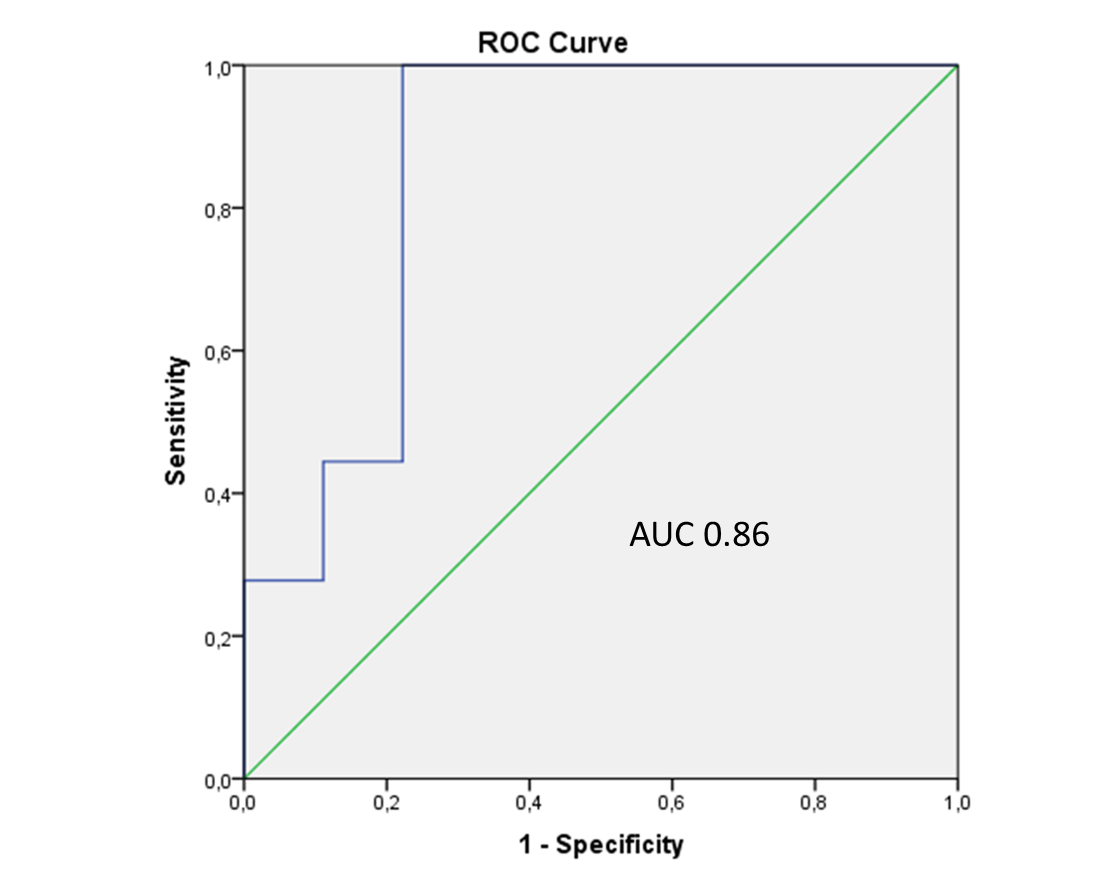

Supplement: S1 Fig — (TIFF) [file pone.0175274.s002.tiff]
